# Understanding and Managing Fracture Risk in Patients With Cancer: A Literature Review

**DOI:** 10.7759/cureus.83082

**Published:** 2025-04-27

**Authors:** Efstathios Kourtis, Konstantinos Zygogiannis, Ilias Fanourgiakis, Dimitrios Koulalis, Konstantinos D Stathopoulos

**Affiliations:** 1 Third Orthopedic Department, KAT General Hospital, Athens, GRC; 2 Scoliosis and Spine Department, KAT General Hospital, Athens, GRC; 3 Orthopedics and Traumatology Department, Attikon University Hospital, Athens, GRC; 4 Metabolic Bone Diseases, School of Medicine, National and Kapodistrian University of Athens, Athens, GRC

**Keywords:** bone metastasis, cancer, diagnostic protocol, fracture risk, management options

## Abstract

One of the most important complications of cancer and its treatments is the increased fracture risk. Fractures can have a significant impact on the patient's quality of life and may be associated with morbidity, mortality, and reduced functional status. The present study aims to investigate the mechanisms underlying the increased risk of fractures in cancer patients, the effect of cancer treatments on fracture risk, and strategies to prevent fractures in this population. This is a literature review using the PubMed - National Center for Biotechnology Information (NCBI), Web of Science, Cochrane Library, Scopus, and electronic internet databases. The search was based on the keywords "fracture risk" AND "cancer", NOT ("osteoporosis" OR "osteoporotic"). Inclusion criteria were clinical studies assessing fracture pathogenesis and prevention, along with fracture risk estimation in cancer patients. Non-human studies, pediatric studies, non-English studies, editorials, and study protocols were excluded. After the application of inclusion and exclusion criteria, 146 studies were finally included. Fracture risk is particularly increased in patients with malignancies. This is due to the direct effect of cancer cells on bone metabolism, the existence of cancer-related factors (bone metastases, hypercalcemia, malnutrition, and increased risk of falls), coexisting diseases (osteoporosis, diabetes mellitus, and rheumatoid arthritis), and the side effects of anticancer treatments (chemotherapy, radiotherapy, and hormone therapy). Fracture risk assessment is based on the measurement of bone mineral density (DXA), the use of the Fracture Risk Assessment Tool (FRAX), laboratory tests (measurement of calcium, phosphorus, vitamin D, alkaline phosphatase, parathyroid hormone, and biomarkers of bone metabolism), and imaging methods (X-rays, computed tomography, magnetic resonance imaging, and PET/CT of bones). To reduce fracture risk in cancer patients, lifestyle changes (exercise, smoking cessation) and anti-osteoclastic drugs such as bisphosphonates and denosumab are administered. Fracture risk in cancer patients is influenced by various factors, including the type of cancer, stage of disease, cancer treatments, bone health status, and presence of bone metastases. Overall, fracture risk in cancer patients is multifactorial and requires comprehensive evaluation and management to optimize bone health and quality of life.

## Introduction and background

Cancer is a disease that affects millions of people worldwide, with more than 10 million associated deaths globally [1]. One of the most important complications of cancer and its treatments is the increased fracture risk [1]. Fractures can have a significant impact on the patient's quality of life and may be associated with morbidity, mortality, and reduced functional status. Fracture risk is higher in patients with malignancies than in the general population and remains increased for up to 10 years [2]. Cancer patients have an estimated 6% cumulative incidence of major fracture over a six-year follow-up [3]. Fracture risk is 9-fold higher in multiple myeloma patients [4]. This increased risk is thought to be due to a combination of factors, including the direct effects of cancer on bone metabolism, bone loss due to cancer treatments, and a higher incidence of falls in cancer patients [5].

In the general population, the impact of fractures on morbidity and mortality is multifaceted and depends on various factors, such as age, sex, and site of fracture. Fractures may lead to up to a 4-fold increase in morbidity and mortality depending on the type of fracture, its location, the age and overall health of the individual, and the quality of medical care received [6]. Similarly, fractures in cancer patients can significantly impact morbidity and mortality, often intertwined with the underlying cancer and its treatment. The risk of mortality increases 2.5-fold after a major fracture and 3.5-fold after a hip fracture within one year in patients with cancer [3]. Postmenopausal breast cancer patients with a history of fractures, especially of the hip, are more likely to die of any cause [7]. Men with prostate cancer have a 2.4-fold higher risk of fracture requiring hospitalization in comparison with the general population [8].

Several types of cancer have a preference for specific bones and types of fractures. Metastatic cancers, such as breast, lung, prostate, and kidney cancers, can spread to the vertebrae, leading to compression or pathological fractures. Breast cancer commonly metastasizes to the long bones of the arms and legs. Pelvic bones can be affected by metastatic cancers originating from various primary sites, leading to fractures. Cancer-related fractures of the ribs can occur due to metastasis from primary cancers such as breast, lung, or prostate cancer [9]. The present study aims to investigate the mechanisms underlying the increased risk of fractures in cancer patients, the effect of cancer treatments on fracture risk, and strategies to prevent fractures in this population.

Materials and methods

**Figure 1:**
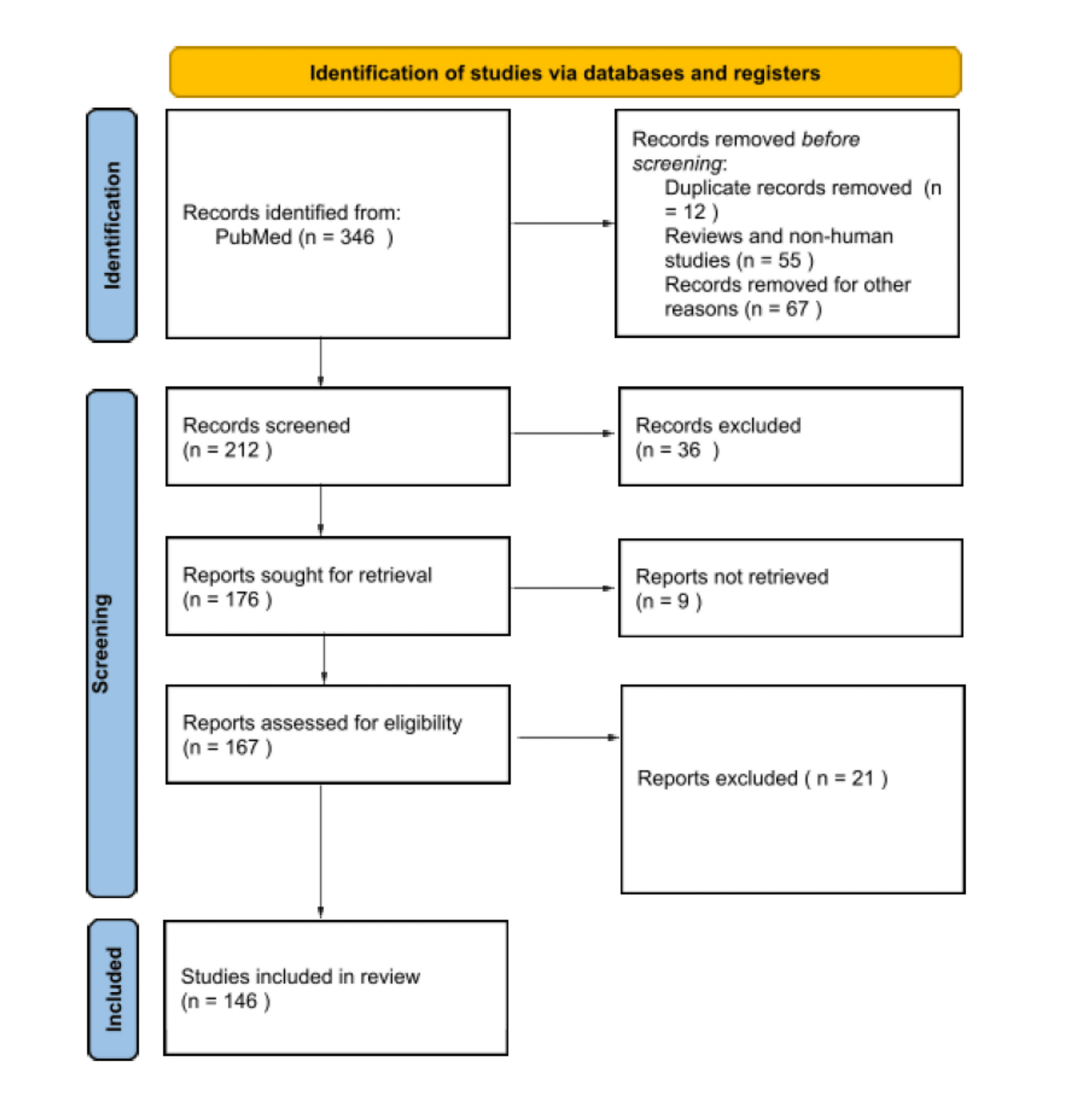
Flowchart with the studies included in the review paper

**Figure 2:**
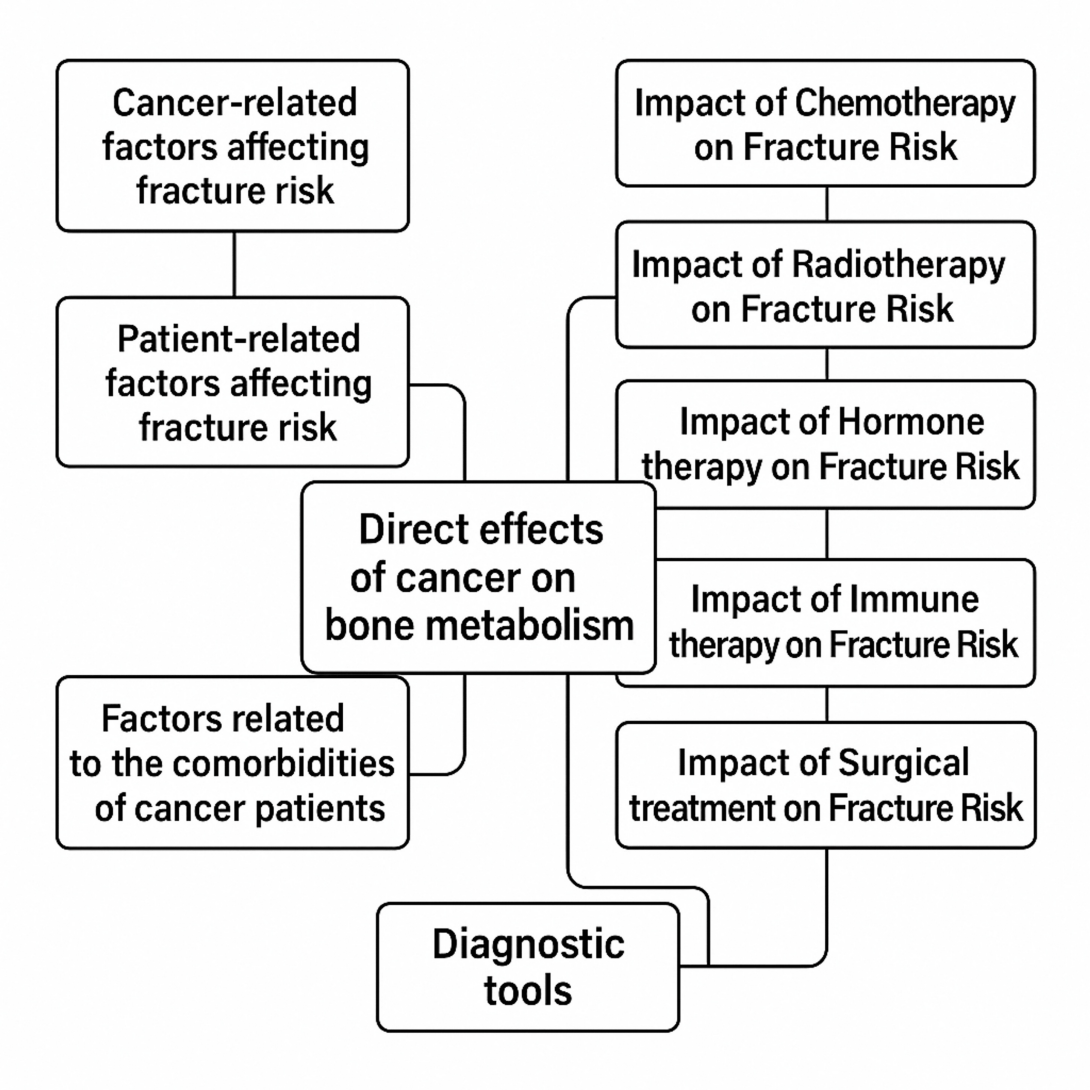
Mind map of review Credit: Image created by the author

This is a systematic literature review using the PubMed - National Center for Biotechnology Information (NCBI), Web of Science, Cochrane Library, Scopus, and electronic internet database. The search was based on the keywords "fracture risk" AND "cancer" NOT ("osteoporosis" OR "osteoporotic"). Inclusion criteria were clinical studies assessing fracture pathogenesis and prevention, along with fracture risk estimation in cancer patients. Non-human studies, non-English studies, editorials, and study protocols were excluded. Studies involving cancer in pediatric populations were excluded. After careful evaluation a total of 146 studies were included. The Preferred Reporting Items for Systematic reviews and Meta-Analyses (PRISMA) guidelines and information regarding the selected studies are demonstrated in Table 1 and Figure 1 while a map graphic of our review methodology is demonstrated in Figure 2.

**Table 1:** Table with study information FRAX: Fracture Risk Assessment Tool

Review in text order	No. of Studies	Reference	Type of study	Year range of studies
Direct effects of cancer on bone metabolism	4	[9-12]	Prospective cohort, retrospective, case control	2005-2023
Cancer-related factors affecting fracture risk	14	[4,9,11,13-23]	Nationwide population-based study, cohort, case study, review	1992-2021
Patient-related factors affecting fracture risk	2	[24-25]	Cohort study	2014, 2016
Factors related to the comorbidities of cancer patients	4	[26-29]	Prospective cohort, meta-analysis, review	2013-2018
Impact of chemotherapy on fracture risk	16	[30-45]	Retrospective cohort, experimental study, cohort, review, retrospective cohort	1990-2023
Impact of radiotherapy on fracture risk	24	[46-64]	Volumetric dosimetry analysis, retrospective cohort, cohort, review, observational, real-world observational study, population-based study	2015-2023
Impact of hormone therapy on fracture risk	35	[25,28,60,65-96]	Randomized controlled study, population-based analysis, nationwide study, cohort, retrospective, review, and meta-analysis	2002-2024
Impact of immune therapy on fracture risk	1	[97]	Exploratory analysis	2022
Impact of surgical treatment on fracture risk	7	[98-104]	Cohort, epidemiological, nationwide study	1996-2017
FRAX Tool	4	[105-108]	Cohort, meta-analysis	2012-2018
Bone mineral density measurement	10	[26,109-117]	Cross-sectional study, cohort, guidelines, review	1998-2024
Laboratory tests	4	[104,118-120]	Cross-sectional study, randomized, cohort	2013-2021
Imaging tests	15	[121-135]	Cross-sectional study, experimental, cohort, randomized trial	2014-2023
Scores	3	[134-137]	Cohort, experimental	2014-2023
Lifestyle modifications	4	[138-141]	Prospective, review, clinical trial	2005-2021
Pharmacological interventions	9	[10,114,123,138,142-146]	Randomized controlled trial, review, clinical trial, cohort	2005-2024

## Review

Pathophysiology

Direct Effects of Cancer on Bone Metabolism

Cancer can directly affect bone metabolism by promoting bone resorption or inhibiting bone formation or both [9]. Increased bone resorption occurs when cancer cells produce growth factors, such as transforming growth factor-beta (TGF-β), that stimulate osteoclast activity. This process leads to loss of bone mass and weakening of bone microarchitecture, making bones more susceptible to fracture [10]. On the other hand, certain types of cancer cells can produce factors that inhibit the activity of osteoblasts [9]. This inhibition can lead to a decrease in bone mineral density (BMD) and an increased risk of fractures. In several types of cancer, such as breast cancer, prostate cancer, lung cancer, thyroid cancer, kidney cancer, and multiple myeloma, the disrupted balance between bone formation and bone resorption may lead to both localized bone loss with osteolytic lesions as well as diffuse osteoporosis [11]. There is an association between papillary thyroid carcinoma and a risk of vertebral fractures independent of sex, BMI, glucose metabolism, and BMD, suggesting the importance of fracture risk assessment before thyroid-stimulating hormone (TSH) suppression [12].

Cancer-Related Factors Affecting Fracture Risk

Bone metastases: Bone metastases are a common complication of advanced cancer, particularly breast, prostate, lung, and multiple myeloma. The incidence of bone metastases varies by cancer type, with breast cancer being the most common cause of bone metastases in women and prostate cancer being the most common cause of bone metastases in men. Bone metastases can structurally weaken bones, leading to fractures [13].

Cancer-induced bone disease (CIBD): CIBD is a term used to describe the effects of cancer on the bones. CIBD can lead to bone loss, bone spurs, and fractures. Mechanisms underlying CIBD include increased osteoclast activity, decreased osteoblast activity, and altered bone extracellular substance composition [9].

Hypercalcemia: Malignant hypercalcemia is a condition characterized by abnormally high levels of calcium in the blood, resulting from cancer-related bone destruction or cancer-related factors such as parathyroid hormone-related protein (PTHrP) secretion. Hypercalcemia can weaken bones, leading to fractures [14]. Hypercalcemia is an independent predictor of overall fracture risk in multiple myeloma patients [4].

Malnutrition: Cancer patients may experience malnutrition due to the cancer itself, cancer treatment, or comorbidities. Malnutrition concerning proteins, calcium, and vitamin D can lead to reduced bone density and increased fracture risk [11,15].

Higher incidence of falls: Cancer patients may be at higher risk of falls, with an estimated prevalence from 30% to 50%, due to various factors, including weakness, sarcopenia, fatigue, visual disturbances, neuropathy, and dizziness caused by cancer treatments [16]. Monoclonal gammapathy results in an increase in falls due to peripheral neuropathy [16]. Falls can lead to fractures and death, especially in patients with weakened bones [17-20]. Factors affecting the risk of falls in cancer patients include age, sex, stage, severity, and type of cancer along with physical and cognitive function, balance/gait, body mass index (BMI), nutrition status, muscle strength, and medication use [21].

Sleep medications: Sleep medication use was common (40%) in breast cancer survivors and was associated with a 33% increased risk of fractures [22].

Prostate-selective alpha antagonists work by blocking the action of alpha-adrenergic receptors in the prostate, leading to relaxation of the smooth muscle tissue in the prostate and bladder neck. This relaxation helps to relieve symptoms associated with prostate cancer, such as urinary hesitancy, frequent urination, and incomplete emptying of the bladder. Their use is associated with a 20% increase of the risk of femoral and skull fractures [23].

Patient-Related Factors Affecting Fracture Risk

In males with lung cancer, smoking status and chronic obstructive pulmonary disease were independently associated with BMD, corrected for age and BMI [24]. Fractures are more common in current smokers, who also have lower bone density [24]. According to Schmidt et al., current smoking status, BMI, dementia, and prescription of corticosteroids have significant impacts on fracture risk [25].

Factors Related to the Comorbidities of Cancer Patients

Osteoporosis: Osteoporosis is a common comorbidity in cancer patients, especially in postmenopausal women and men receiving hormone therapy for prostate cancer. Osteoporosis can lead to reduced BMD and increased fracture risk [26].

Diabetes mellitus: Diabetes mellitus is a common comorbidity in cancer patients. Diabetes mellitus can lead to reduced BMD and increased fracture risk. Mechanisms underlying bone loss associated with diabetes include increased formation of advanced glycation end products (AGEs), decreased insulin-like growth factor-1 (IGF-1) levels, and increased inflammatory status [27-28].

Rheumatoid arthritis: Rheumatoid arthritis is a chronic inflammatory disease that may increase the risk of fracture in cancer patients [29].

Impact of chemotherapy on fracture risk

Chemotherapy is one of the main treatments used to treat various types of cancer, but it can also have harmful effects on bone health. Chemotherapy is a systemic treatment that targets rapidly dividing cancer cells. However, it can also affect healthy cells, including bone cells [30]. Chemotherapy-induced bone loss (CIBL) is a common complication of chemotherapy, particularly in postmenopausal women and men receiving hormone therapy for prostate cancer. It refers to the accelerated loss of BMD and increased risk of fractures that can occur as a side effect of certain chemotherapy medications. It is more common in patients receiving aromatase inhibitors (AIs), glucocorticoids, and platinum-based chemotherapy [31].

Chemotherapy can cause bone loss and increase fracture risk by several mechanisms, such as [32-33]:

Direct Cytotoxicity to Osteoblasts

Chemotherapy can have a detrimental effect on osteoblasts, leading to reduced bone formation. For example, drugs like methotrexate can inhibit folate metabolism, which is essential for osteoblast function and bone formation [34]. Platinum-based drugs like cisplatin can induce oxidative stress and DNA damage in osteoblasts, leading to cell death and impaired bone formation [35].

Stimulation of Osteoclasts

Chemotherapy affects osteoclast activity, resulting in modified increased bone resorption. Chemotherapy drugs, particularly cytotoxic agents, can suppress bone marrow function, leading to a decrease in the production of blood cells, including osteoclast precursor cells. This suppression can indirectly affect osteoclast activity, leading to reduced bone resorption [36].

Indirect Effects on Bone Cells

Chemotherapy can affect the production of hormones that regulate bone metabolism, such as estrogen, testosterone, and parathyroid hormone. Chemotherapy can damage or suppress the function of the gonads, leading to gonadal dysfunction and hormonal imbalances. For example, chemotherapy-induced ovarian failure in premenopausal women can result in decreased estrogen production, which is associated with accelerated bone loss and an increased risk of osteoporosis. Similarly, chemotherapy-induced testicular dysfunction in men can lead to decreased testosterone levels, which also contribute to bone loss and osteoporosis [37-38].

Nutritional Deficiencies

Chemotherapy-induced gastrointestinal toxicity may lead to malabsorption of calcium and vitamin D, which are essential for bone health [39].

Increased Frequency of Falls

Chemotherapy leads to an increased risk of falls, resulting in an increased fracture risk [40-41]. Chemotherapy drugs can cause muscle weakness, fatigue, dizziness, and cognitive impairment as side effects, impairing balance and coordination and increasing the risk of falls. Some chemotherapy drugs can cause peripheral neuropathy, affecting sensation and proprioception, making it more difficult for patients to maintain balance and avoid falls [41].

In premenopausal women with breast cancer, chemotherapy is associated with increased fracture risk by inducing early menopause. Premenopausal women with breast cancer under chemotherapy had significantly decreased lumbar spine BMD in comparison to age-matched patients not receiving chemotherapy. The difference in BMD was attributed to the high incidence of chemotherapy-induced menopause [42]. It has been calculated that chemotherapy-induced menopause may hasten fractures by approximately 10 years in premenopausal women with breast cancer [42]. Indeed, CIBL in patients with chemotherapy-induced premature menopause results in a marked decrease in BMD within a short period of time [43]. Chemotherapy has been found to increase fracture risk in patients with soft tissue sarcomas of the proximal lower extremity and germ cell tumors [44-45].

Impact of radiotherapy on fracture risk

Radiotherapy uses high-energy beams to kill cancer cells and is commonly used in the treatment of various cancers, including breast, prostate, lung, and bone metastases. Radiation can damage bone cells, weakening bones, leading to decreased bone density and increased fracture risk, especially in the radiation field. The risk of fractures is higher in bones exposed to high doses of radiation, such as the spine and hip [46]. The mechanism of radiation-induced bone loss includes [47]:

Direct Damage to Bone Cells

Radiation can damage osteoblasts and osteoclasts, leading to decreased bone formation and increased bone resorption, altering the composition of bone extracellular matrix. Direct exposure to radiation can cause DNA damage, cell death, and disruption of cellular functions in osteoblasts, osteoclasts, and bone marrow stromal cells [48].

Vascular Damage

Radiation can damage blood vessels in bones, leading to reduced blood supply and oxygenation, which can further weaken bones [48].

Pelvic radiation has been associated with a 9.4% incidence of pelvic insufficiency fractures, especially in women above 50 years of age with gynecological or anal cancers. Risk factors include osteoporosis, menopause, and diabetes mellitus [49]. In these patients, the fracture risk is especially increased in women at 2-4 years after pelvic radiotherapy [50]. Women with pelvic cancer treated with pelvic radiation, in comparison to women with pelvic cancer not treated with pelvic radiation, had a 2-fold higher pelvic fracture risk [51]. Short-course preoperative radiotherapy increases pelvic fracture risk in rectal cancer [52].

Stereotactic body radiation therapy of 27 Gy dose is associated with a 6% fracture incidence in non-spine bone metastases, especially in females with lytic lesions [53]. When 30-35 Gy are used, the fracture incidence reaches 8.5% [54]. According to a study by Ito et al., stereotactic body radiotherapy at a 30-35 Gy dose, for curative intent, should not be contraindicated in long bone oligometastasis because fractures do not directly contribute to life expectancy [55]. Vertebral compression fractures are a common complication after stereotactic body radiation therapy, with a crude incidence of 12.1%. It is recommended that the maximum point dose should be <24 Gy [56]. Multifraction stereotactic body radiation therapy for metastatic spinal disease results in a low vertebral fracture risk in comparison with single-fraction stereotactic body radiation therapy [57].

External-beam radiation therapy combined with limb-sparing surgery is feasible in elderly patients with primary soft tissue sarcomas of the extremities, with a slight increase in fracture risk [58]. 50 Gy of radiation therapy has been found to increase fracture risk in patients with soft tissue sarcomas of the proximal lower extremity [44]. In patients with soft tissue sarcoma, the cumulative risk of femur fracture in patients treated with intensity-modulated radiation therapy was 6.7% [59].

Radium-223 dichloride is a radiopharmaceutical used in the treatment of metastatic castration-resistant prostate cancer (mCRPC). It mimics calcium and tends to accumulate in areas of increased bone turnover, such as sites of bone metastases. Once administered intravenously, radium-223 is taken up by the bone metastases, where it emits alpha particles, thereby delivering targeted radiation to the cancer cells in the bone. Its use has been associated with an increase in fracture risk [60-62].

In breast cancer patients, radiotherapy has been associated with a 5.5-fold increased fracture risk [63]. In radiated femoral metastases, fractures have been reported to occur a median of 4.4 months after radiotherapy [64].

Impact of hormone therapy on fracture risk

Hormone therapy is a treatment that targets the hormones that fuel cancer growth. It is commonly used in breast and prostate cancer. Hormone therapy, particularly in the form of androgen deprivation therapy (ADT) for prostate cancer and AIs for breast cancer, can cause bone loss and increased fracture risk [65-66]. Hormone therapy can cause bone loss and increase the risk of fractures by several mechanisms, including:

Decreased Levels of Estrogen and Testosterone

Estrogen and testosterone are key hormones for bone health. Hormone therapy can reduce these hormones, leading to decreased bone density and increased fracture risk [66-67].

Increased Bone Resorption

Hormone therapy can increase osteoclast activity, leading to bone resorption and decreased bone density.

Tamoxifen, commonly used in the treatment of hormone receptor-positive breast cancer, belongs to a class of drugs known as selective estrogen receptor modulators (SERMs). Tamoxifen works by blocking the effects of estrogen in breast tissue, and it is often prescribed for both the treatment of breast cancer and as a preventive measure in certain high-risk populations. The relationship between tamoxifen and fracture risk is complex, and the available evidence suggests a nuanced picture [68]. Most studies suggest that tamoxifen may have a positive impact on bone density, particularly in postmenopausal women. Tamoxifen has been associated with a 25-50% reduction in the risk of osteoporosis and osteoporotic fractures [69-70].

AIs, such as anastrozole, exemestane, and letrozole, are a class of medications commonly used in the treatment of hormone receptor-positive breast cancer in postmenopausal women. Unlike tamoxifen, AIs work by blocking the enzyme aromatase, which is responsible for converting androgens into estrogen in postmenopausal women [63]. By reducing estrogen levels, AIs aim to deprive hormone receptor-positive breast cancer cells of the estrogen they need to grow and divide. AIs are commonly used as adjuvant therapy in postmenopausal women with early-stage hormone receptor-positive breast cancer to reduce the risk of recurrence. Guidelines recommend considering AI therapy as initial adjuvant treatment or after 2 to 3 years of tamoxifen therapy. Adjuvant AI therapy is typically administered for 5 to 10 years, depending on the specific clinical scenario and patient factors [71]. In young women with breast cancer, AI administration has been associated with an 8-fold increased fracture risk [63]. AIs have been associated with a 2-4 times increased rate of reduction in BMD in postmenopausal women. Numerous studies have suggested an increased fracture risk, particularly at sites such as the spine, hip, and wrist, in postmenopausal women taking AIs [72-78]. The use of anastrozole is associated with a higher fracture risk [79]. A recent meta-analysis estimated a relative risk of 1.35 for all osteoporotic fractures, 1.18 for hip fractures, 1.84 for vertebral fractures, and 1.18 for non-vertebral fractures in patients with breast cancer on AI therapy [80]. The increased fracture risk may be attributed to the reduction in estrogen levels and subsequent bone loss. Fracture risk may increase with prolonged use of AIs. BMD tends to normalize after cessation of AI therapy [81]. Total nonvertebral fracture risk is higher for users of AIs compared with tamoxifen [82]. Fracture rates in the first years after cessation of hormonal therapy for breast cancer were 12% and 15% for pre- and postmenopausal women, respectively. The most common fractures in postmenopausal women were hip and major fractures [83]. Fat body mass may be associated with fragility-related fractures in patients with breast cancer who undergo AI therapy [84]. A synergism between lean body mass and high-fat body mass has been observed in predicting the risk of vertebral fractures in women with early breast cancer undergoing AIs [85]. Compliance with AI treatment in women with breast cancer is associated with a clear increase in fracture risk [25].

Gonadotropin-releasing hormone (GnRH) agonists, such as goserelin, leuprolide, abarelix, and buserelin, are medications that are often used in the treatment of prostate cancer, breast cancer, and certain gynecological disorders [86-87]. They act by initially stimulating the release of gonadotropins, leading to an increase in sex hormone levels. However, with continued use, GnRH agonists ultimately suppress the production of sex hormones, including testosterone in men and estrogen in women [88]. The impact of GnRH agonists on bone health and fracture risk is primarily related to the reduction of estrogen and/or testosterone levels. GnRH agonists are associated with approximately 10% decline in BMD at the spine after two years, and when used in combination with AIs, 17% decline in BMD at the spine after three years [89-90]. As a result, individuals treated with GnRH agonists may be at an increased risk of fractures.

ADT is commonly used in the treatment of prostate cancer. It aims to reduce levels of testosterone, which can fuel the growth of prostate cancer cells. Testosterone plays a crucial role in maintaining bone density in men. Reduced testosterone levels (up to <5% of normal) due to ADT can lead to accelerated bone turnover and a decline in BMD, increasing fracture risk, particularly in weight-bearing bones such as the hip and spine [28,91-92]. ADT results in approximately a 4% decrease in spine BMD within 12 months of treatment [93-94]. ADT has been associated with a 39% increased overall fracture risk and a 55% risk for fracture requiring hospitalization [95]. The number of new bone fractures occurring on or after ADT-investigational agent treatment has been reported to be 4.6 per 1000 person-years [96].

Abiraterone acetate is a medication used in the treatment of prostate cancer, specifically mCRPC. It is also sometimes used in combination with prednisone or prednisolone. Abiraterone acetate works by inhibiting the production of androgens within the body. It blocks CYP17A1, an enzyme involved in the production of androgens in the adrenal glands and the tumor itself. By reducing the levels of androgens in the body, abiraterone acetate can slow down the progression of prostate cancer and alleviate symptoms associated with the disease. Its use is generally safe and has been associated with a very low fracture risk [60].

Impact of immunotherapy on fracture risk

Immune checkpoint inhibitors are a class of medications used in cancer immunotherapy. They act by targeting specific proteins, known as checkpoint molecules, that regulate immune responses. By blocking these checkpoint molecules, immune checkpoint inhibitors can help the immune system recognize and attack cancer cells more effectively. Immune checkpoint inhibitors have been approved for the treatment of various types of cancer, including melanoma, non-small cell lung cancer, renal cell carcinoma, bladder cancer, Hodgkin lymphoma, and others [97]. According to a study by Pantano et al., a three-month course of immune checkpoint inhibitors led to a significant increase of CTX-I with a concomitant decreasing trend toward the reduction of PINP, suggesting a relationship between immune checkpoint inhibitors and increased osteoclast activity and potential fracture risk [97].

Impact of surgical treatment on fracture risk

Surgery, especially on the hip and spine, can increase the risk of fracture in cancer patients. Surgery can weaken bones and alter bone biomechanics. Surgical procedures such as gastrectomy for gastric cancer and hysterectomy with bilateral oophorectomy for gynecological cancers can also lead to deleterious effects on bone health [98-99].

Hibler et al. reported 9% BMD loss at the lumbar spine and 6% BMD loss at the hip in premenopausal women with breast cancer undergoing risk-reducing bilateral salpingo-oophorectomy over 18 months [100]. Women aged 45-55 years with early menopause and early bilateral salpingo-oophorectomy had lower vertebral and femoral BMD in comparison with women who underwent natural menopause [101]. Effects on fracture risk after premenopausal bilateral salpingo-oophorectomy are inconsistent. One cohort study observed increased fracture risk in the distal wrist and spine but not in the hip, while another study did not detect increased risk for non-vertebral fractures [102-103]. Five years after bilateral salpingo-oophorectomy, bone turnover biomarkers were higher than age-matched reference values, predicting a higher fracture risk [104].

Diagnosis

Fracture risk assessment is essential in cancer patients to identify patients at high risk of fracture and to implement appropriate preventive measures. Several tools are available to assess fracture risk, including the Fracture Risk Assessment Tool (FRAX) and measurement of BMD.

FRAX Tool

The FRAX is a widely used tool that estimates the 10-year probability of hip fracture and major osteoporotic fractures. It takes into account many factors, including age, sex, weight, height, smoking, alcohol consumption, family history of hip fracture, individual history of fracture, and the presence of comorbidities such as rheumatoid arthritis, secondary osteoporosis, and diabetes [105-106]. Although FRAX does not directly assess falls, it has been observed to predict falls, especially in elderly men, and may capture some of this associated fracture risk [107-108].

BMD measurement

Bone densitometry is a non-invasive test that measures bone density. It is an essential tool for diagnosing osteoporosis and assessing fracture risk in cancer patients. BMD is usually measured using dual-energy X-ray absorptiometry (DXA) [109]. A BMD test measures the amount of bone mineral in a specific area, usually the hip, spine, or wrist, and is compared to the average BMD of young healthy women to produce T-scores that are used for the diagnosis of osteoporosis. It can diagnose osteoporosis and predict fracture risk. For osteopenia, the T-score typically falls between -1.0 and -2.5 standard deviations (SD) below the average BMD of a young adult reference population. Osteoporosis is defined as when the T-score is < -2.5 SD, indicating a higher risk of fracture [26]. However, these tools may not be applicable to all cancer patients, especially those with bone metastases. In these patients, bone imaging techniques such as radiographs, computed tomography (CT) scans, magnetic resonance imaging (MRI), or bone scintigraphy may be more appropriate to assess the extent of bone involvement and identify any impending fractures [110-111].

Although BMD is a good predictor for bone strength, it does not take into consideration bone size, geometry, and microarchitecture. This is the reason why a lot of women with fragility fractures have no osteoporosis as measured by DXA and may be termed as suffering merely from osteopenia [112-113]. Periodic BMD measurement is recommended for all women with breast cancer under hormone therapy [114]. Any annual BMD decrease of 10% or more in comparison with pretreatment levels is suggestive of initiation of antiosteoporotic treatment [115]. In multiple myeloma, BMD has been found to be lower than age- and sex-matched controls [116]. BMD is lower at the spine and is associated with female sex, duration of disease, and number of IgA strains [117].

Laboratory tests

Laboratory tests are necessary to assess the patient's bone metabolism and to identify any underlying metabolic disorders that may contribute to bone loss or bone metastases [118].

Serum Calcium and Phosphorus Levels

Measurement of serum calcium and phosphorus levels is necessary to assess the patient's bone mineral metabolism. Abnormal levels of calcium and phosphorus may indicate bone loss or bone metastases [119].

Serum Alkaline Phosphatase and Parathyroid Hormone Levels

Alkaline phosphatase is an enzyme produced by osteoblasts. Elevated alkaline phosphatase levels may indicate increased bone metabolism, which may be a finding of bone metastases or osteoporosis. Parathyroid hormone is a hormone produced by the parathyroid glands that regulates calcium and phosphate metabolism. Abnormal levels of parathyroid hormone may indicate an underlying metabolic disorder that may contribute to bone loss or bone metastases [120].

Serum Vitamin D Levels

Vitamin D is essential for calcium absorption, and its deficiency can contribute to bone loss or bone metastases. Serum vitamin D levels can help identify patients who may need supplementation [120].

Markers of bone resorption (CTX, TRAPc5b) and bone formation (osteocalcin, bone ALP, PINP) [104].

Imaging tests

Imaging studies are essential to diagnose bone metastases and to assess the extent of bone destruction and risk of fracture. Imaging studies commonly used to diagnose increased fracture risk in cancer patients include [121]:

X-ray

An X-ray is a non-invasive test that uses low-dose radiation to produce images of the bones. Radiography is commonly used to diagnose fractures and to assess the extent of bone destruction in patients with bone metastases [122]. Axial cortical involvement ≥30 mm had a 5.3 times higher fracture risk [122]. Radiographic screening for atypical femoral fractures could be relevant in patients who have been on long-term oncologic bone-modifying agents [123]. X-rays provide direct visualization of bone structures, allowing the assessment of the integrity and alignment of the vertebral bodies. Fractures typically appear as disruptions or discontinuities in the normal contour of the vertebral body on X-ray images.

Computed Tomography

A CT scan is a non-invasive test that uses X-rays and computer analysis to produce detailed images of the bones. CT is useful for the diagnosis of bone metastases and for estimating the extent of bone destruction [124-127]. Biomechanical CT (BCT) is a radiomic technique that measures BMD and bone strength from CT scans. Among men diagnosed with metastatic hormone-sensitive prostate cancer, BCT assessments were strongly correlated with DXA and predicted subsequent pathologic fracture [128]. CT-based rigidity analysis has a small influence on perceived fracture risk [129-130].

Magnetic Resonance Imaging

MRI is a non-invasive test that uses magnetic fields and radio waves to produce detailed images of bones and soft tissue. MRI is useful for diagnosing bone metastases, spinal cord compression, and other complications of bone loss [131].

Fluorodeoxyglucose positron emission tomography/CT (FDG PET/CT) is a combined imaging technique that merges the functional information obtained from PET with the anatomical details provided by CT scans. FDG PET/CT may help for the assessment of fracture risk in cancer patients [132].

Radiomic variables are quantitative features extracted from medical images using advanced image processing techniques. These variables capture information about the shape, texture, intensity, and spatial relationships of structures within the images. Radiomic features, including a bone density score and radiomic signatures from the ribs, can be used to stratify risk of rib fracture after stereotactic body radiation therapy for early-stage non-small cell lung cancer [133-135].

Scores

The Mirel's Score is a scoring system used to assess the risk of pathological fractures in patients with metastatic bone disease. It takes into account several factors related to the tumor and its effect on bone integrity, such as the location of the lesion, the size of the lesion, the type of lesion, and the presence of pain. Based on these factors, each parameter is scored, and the scores are then summed to give an overall Mirel's Score [136-137]. The total score ranges from 0 to 12, with higher scores indicating a greater risk of fracture. A Mirels score of ≥ 8 points had the best predictive profile for fractures at humeral metastases [137].

The Spinal Instability Neoplastic Score (SINS) is a scoring system used to assess the stability of the spine in patients with metastatic spinal tumors. It takes into account the location of the tumor, the type of spinal involvement, the degree of vertebral body collapse, the presence of posterolateral involvement, pain severity, and neurological deficit. SINS is demonstrated to be a useful tool to assess fracture risk in patients with bone metastases after radiation therapy [136].

The bone strength score is based on a CT-based patient-specific finite element computer model that objectively calculates bone strength. It may be a useful tool for the assessment of fracture risk in femoral bone metastases [134]. A prognostic score assessing eight variables (age, race, hormone treatment, Elixhauser score, anxiety, Parkinson's, fall-inducing medications, and disability status) may predict fracture risk in prostate cancer patients [135].

Prevention

There are several strategies that can be used to prevent fractures in cancer patients, including lifestyle modifications, pharmacological interventions, and monitoring of bone health.

Lifestyle modifications

Lifestyle modifications can improve bone health and reduce fracture risk in cancer patients. These modifications include [138]:

Adequate Intake of Calcium and Vitamin D

Adequate intake of calcium and vitamin D can improve bone density and reduce the risk of fracture. For cancer patients, the recommended daily intake of calcium is 1200 mg for adults over 50 and 800-1000 IU of vitamin D per day.

Regular Exercise

Weight and resistance exercises can improve bone health and reduce the risk of fracture. Exercise may also alleviate cancer-related fatigue and improve overall quality of life [139-140]. Combined impact loading and resistance exercise attenuates bone loss at the spine and reduces fracture risk in prostate cancer patients undergoing ADT [141].

Smoking Cessation

Smoking is a risk factor for osteoporosis and fractures. Quitting smoking can improve bone health and reduce fracture risk [139].

Pharmacological interventions

Pharmacological interventions are an essential component of fracture prevention in cancer patients. The following are some of the pharmacological interventions that can help prevent bone loss and reduce fracture risk:

Bisphosphonates

Bisphosphonates are a class of drugs that can help prevent bone loss and fractures by inhibiting osteoclast activity. Bisphosphonates are commonly used to treat osteoporosis and bone metastases in cancer patients [114,142-143]. Risedronate preserves BMD and may attenuate loss of bone microarchitecture over two years in breast cancer survivors on AIs [144]. However, according to Drieling et al., long-term (> 8 years) use of bisphosphonates is an independent risk factor for fractures in postmenopausal women [145]. Prolonged use of zoledronate in cancer patients could predispose them to atypical femoral fractures [123]. Zoledronic acid (Zometa) is the bisphosphonate of choice for the management of malignancy-associated bone disease. Its efficacy in reducing skeletal-related events and preventing bone resorption makes it a cornerstone of supportive care in oncology. The standard dosage regimen is 4 mg, which can be administered multiple times per year in patients with multiple secondary metastases. In the absence of metastatic disease and the absence of specific guidelines for a given neoplastic condition, a yearly administration of 5 mg may be considered to maintain bone health and reduce the risk of skeletal complications. However, in malignancies with established protocols, such as breast cancer, zoledronic acid is typically administered every six months. Treatment schedules should be individualized based on disease status, patient risk factors, and emerging clinical guidelines.

Denosumab

Denosumab is a monoclonal antibody that can help prevent bone loss and fractures. It works by inhibiting the activity of RANKL, which is a protein that stimulates osteoclasts. Denosumab is commonly used to treat osteoporosis and bone metastases in cancer patients [143]. Prolonged use of denosumab in cancer patients could predispose them to atypical femoral fractures [123].

Hormone Therapy

Estrogen or testosterone replacement therapy can help prevent bone loss and fractures in cancer patients. Hormone therapy is usually reserved for patients who have low hormone levels and are at increased risk of bone loss [10,142,146].

Calcitonin

Calcitonin is a hormone that can help prevent bone loss and fractures. It works by inhibiting the activity of osteoclasts. Calcitonin is commonly used for bone pain management in cancer patients who are unable to tolerate other drugs such as bisphosphonates or denosumab [138].

## Conclusions

Fracture risk in cancer patients is influenced by various factors, including the type of cancer, stage of disease, cancer treatments, bone health status, and presence of bone metastases. Fracture risk is particularly increased in patients with malignancies. This is due to the direct effect of cancer cells on bone metabolism, the existence of cancer-related factors (bone metastases, hypercalcemia, malnutrition, increased risk of falls), coexisting diseases, and side effects of anticancer treatments. Fracture risk assessment is based on the measurement of bone density, the use of the FRAX tool, laboratory tests, and imaging methods. To reduce fracture risk in cancer patients, lifestyle modification and anti-osteoclastic drugs such as bisphosphonates and denosumab are needed. Overall, fracture risk in cancer patients is multifactorial and requires comprehensive evaluation and management to optimize bone health and quality of life.
